# The delayed cancer treatment and economic inequality in Korea: results of common cancers by the time-to-surgery

**DOI:** 10.4178/epih.e2025056

**Published:** 2025-09-27

**Authors:** Noorhee Son, Woo-Ri Lee, Dong-Woo Choi, Kyu-Tae Han

**Affiliations:** 1 HIRA Research Institute, Health Insurance Review & Assessment Service (HIRA), Wonju, Korea; 2 Division of Cancer Control & Policy, National Cancer Control Institute, National Cancer Center, Goyang, Korea; 3 Cancer Big Data Center, National Cancer Control Institute, National Cancer Center, Goyang, Korea

**Keywords:** Health services accessibility, Neoplasms, Healthcare disparities, Delivery of health care, Mortality

## Abstract

**OBJECTIVES:**

Growing concerns regarding the concentration of cancer treatment in the capital city in Korea have raised questions about equitable access to timely and optimal patient care. In this study, we evaluated the impact of time-to-surgery (TTS) on healthcare utilization and outcomes, with the goal of providing policy recommendations for effective quality assessment of cancer care.

**METHODS:**

This retrospective cohort study analyzed data from 2011 to 2021 obtained from National Health Insurance Service claims. A generalized estimating equation and a Cox proportional hazards model were applied to assess the effects of TTS on length of stay (LOS), medical costs, and 5-year mortality among patients diagnosed with lung, liver, and colorectal cancers. Subgroup analyses were conducted based on patients’ baseline economic status.

**RESULTS:**

Among patients who underwent surgical treatment for lung, liver, or colorectal cancer, 20.4%, 11.4%, and 11.4% experienced treatment delays, respectively. Regardless of cancer type, longer TTS was associated with prolonged LOS and higher medical costs. Moreover, patients with extended TTS demonstrated an increased risk of 5-year mortality. Disparities by income level were evident, with greater differences observed in the lower-income group.

**CONCLUSIONS:**

This study highlights the importance of timely surgical treatment for patients with cancer, particularly in relation to income-based disparities. These findings emphasize the need to improve Korea’s concentrated cancer care delivery system to enhance healthcare efficiency and address health literacy gaps affecting treatment by income level.

## GRAPHICAL ABSTRACT

[Fig f3-epih-47-e2025056]

## Key Message

• Among surgical patients, approximately 10-20% experienced treatment delays.

• Longer time-to-surgery (TTS) was linked to prolonged length of stay (LOS), higher medical costs, and an increased 5-year mortality risk.

• Income-level disparities were evident, with more pronounced adverse differences in lower-income groups.

## INTRODUCTION

Lung, liver, and colorectal cancers pose a major public health burden in Korea, ranking among the top 3 causes of cancer-related mortality and accounting for more than 10% of all cancer deaths [1]. These cancers also impose a substantial disease burden and require intensive healthcare resources [2]. Such cancers often necessitate prompt and active interventions, including surgery or chemotherapy, immediately after diagnosis to improve prognosis [3-6]. Delays in initiating treatment can adversely affect patient outcomes, resulting in higher mortality and increased healthcare utilization. Therefore, timely management of lung, liver, and colorectal cancers is essential for improving survival and reducing avoidable medical costs.

The establishment of a healthcare system that ensures timely and optimal cancer care is central to effective national cancer control. Although previous studies have raised concerns regarding delayed cancer treatment and its outcomes, these issues have not been adequately addressed from a policy perspective [7-9]. In 2021, efforts began to include time-to-surgery (TTS) as a quality measure in cancer care. The Health Insurance Review and Assessment Service (HIRA) announced revisions to its quality assessment (QA) system, shifting focus from surgical outcomes to overall cancer treatment. The results of this revised QA are scheduled to be disclosed after 2024 [10]. Health policies such as HIRA’s QA have explicit objectives. Performance evaluation seeks to enhance care quality by fostering competition and benchmarking among hospitals. Public reporting is intended to guide patients in making hospital choices based on evaluation results. Because cancer care practices vary depending on patient characteristics, the influence of QA on TTS may differ across groups. However, only limited studies have examined TTS and disparities, and previous findings are not representative of all cancer patients in Korea [11,12].

Over recent decades, Korea has made progress in easing economic and regional barriers to cancer care. Despite this, most patients continue to prefer large hospitals in Seoul [13]. This concentration has fueled concerns about patient clustering and treatment delays. According to 2022 disease statistics, more than 70% of newly diagnosed cancer patients in Korea sought care at hospitals in the Seoul metropolitan area [14]. While QA for cancer care has contributed to quality improvement through inter-hospital competition, it has not sufficiently enabled informed decision-making by patients [15]. Unlike broadly accessible health information, such as cancer prevention strategies, specialized knowledge such as TTS may be difficult for patients and caregivers to interpret. Consequently, an optimal system must be developed that aligns cancer care delivery with patient-specific characteristics. In this study, we investigated the effects of TTS delays on healthcare utilization (medical costs and length of stay [LOS]) and mortality among Korean patients with lung, liver, and colorectal cancers. We also examined disparities in TTS and associated outcomes across income groups. Our aim was to provide evidence that can inform national cancer policy and QA frameworks to reduce treatment delays and advance equity in cancer care.

## MATERIALS AND METHODS

### Study design and study population

To investigate the effect of time to surgical treatment on lung, liver, and colorectal cancers, we used customized Korean health information data managed by the National Health Insurance Service (NHIS) from 2011 to 2021. The NHIS oversees the enrollment of the insured and their dependents, who account for approximately 97% of the population, as well as their medical claim records. NHIS claims data are publicly available and may be used for research purposes [16]. In this study, we employed customized data that included information on insurance eligibility, medical treatment, health examinations, medical institutions, and other socio-demographic characteristics. Patients with lung, liver, or colorectal cancer were identified using the International Classification of Diseases, 10h revision (ICD-10) codes in combination with the specific cancer claim code “V193.”

**Figure 1.**
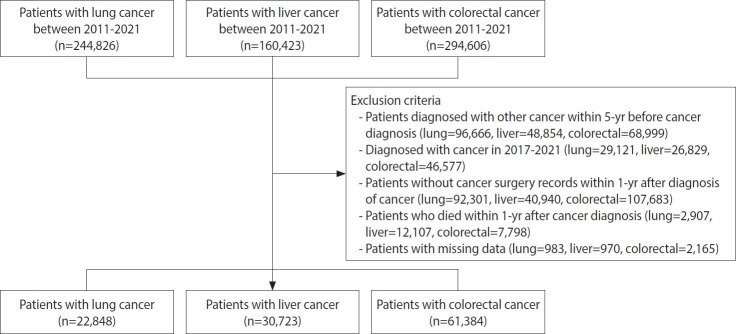
Flowchart of the study population. Flowchart of the study population with lung, liver, and colorectal cancer.

The study population was initially selected based on ICD-10 codes for lung cancer (“C33,” “C34”), liver cancer (“C22”), and colorectal cancer (“C18,” “C19,” “C20”) between 2011 and 2021, with the specific cancer claim code “V193” (lung cancer=244,826; liver cancer=160,423; colorectal cancer=294,606). We excluded patients who had been diagnosed with other types of cancer within 5 years prior to the index diagnosis. To ensure a complete 5-year observational period, we included only patients diagnosed between 2011 and 2016. Patients without surgical treatment records within 1 year of diagnosis and those who died within 1 year of diagnosis were excluded to reduce immortal time bias and heterogeneity. Patients with missing data were also excluded (Figure 1). The final study population comprised 22,848 patients with lung cancer, 30,723 patients with liver cancer, and 61,384 patients with colorectal cancer.

### Variables

#### Outcome variables

The outcome variables were LOS, medical costs, and 5-year mortality. LOS was determined by summing all inpatient records with the cancer-specific claim code “V193.” Medical costs were calculated using the same method. LOS and medical costs were adjusted for the total observational period and standardized to a 5-year value for comparison. Five-year mortality was determined by identifying deaths within 5 years of cancer diagnosis. Participants who died within 5 years were categorized as “died,” and the time to death was used for survival analysis. Participants who survived 5 years or were lost to follow-up were categorized as “survivors,” and their total observation period was recorded. The formulas used in the analysis were as follows:

Length of stay=(Sum of length of stay during 5-year observation period÷total observation period)×1,825

Medical costs=(Sum of medical costs during 5-year observation period÷total observation period)×1,825

5-year mortality=death since the diagnosis of the lung, liver, or colorectal cancer

#### Interesting variable

The primary variable of interest was the delay in waiting time for surgical treatment. Definitions of delay vary across studies [17,18]. In Korea, QA programs have been implemented since 2001 to evaluate and improve the quality of medical services. Recent reforms in the national QA program have emphasized timely surgery for colorectal, stomach, lung, liver, and breast cancers, using a 30-day threshold after diagnosis as a key indicator [19,20]. This benchmark reflects both domestic practice—where nearly 90% of gastric, colorectal, and lung cancer patients undergo surgery within 30 days—and international standards applied in the United Kingdom and other Organization for Economic Cooperation and Development (OECD) countries [21,22]. Based on this policy relevance and clinical evidence, and considering studies indicating poorer outcomes among patients treated after 30 days [23,24], we adopted a 30-day threshold to define surgical treatment delay from the initial hospital visit for lung, liver, or colorectal cancer.

#### Covariates

We incorporated socio-demographic, medical, and healthcare utilization factors as covariates. Sex was categorized as male or female; age groups were defined as <55 years, 55-64 years, 65-74 years, or ≥75 years. Residential area was stratified as Seoul, other metropolitan, or non-metropolitan. Income level was classified as medical-aid, below-median, or above-median. The national health security system consists of mandatory social health insurance and medical-aid. The medical-aid program, funded by government subsidies, supports low-income groups by covering healthcare costs and exempting them from insurance premiums [25]. The medical-aid group is reimbursed under a different rate, resulting in distinct healthcare utilization patterns compared with low-income NHI beneficiaries. The Charlson comorbidity index (CCI) was used to account for comorbid conditions prior to cancer diagnosis, with conditions identified in the year preceding diagnosis and weighted according to Quan et al. [26]. The sum of weights yielded a single comorbidity score. Treatment type was determined based on therapy records within 1 year of diagnosis and categorized as surgical, chemotherapy, or radiotherapy. Patients were then grouped into “only surgery” or “surgery with chemotherapy or radiotherapy.” Additional variables included year of cancer diagnosis, occurrence of other cancer types within 5 years after diagnosis (yes/no), and type of primary treatment institution based on costs within 1 year (tertiary hospital or other).

### Statistical analysis

We first described patient characteristics using frequencies and percentages, followed by comparisons of LOS, medical costs, and 5-year mortality by patient characteristics using the t-test, analysis of variance, and chi-square test. Statistical significance was set at p<0.05. The associations between TTS, LOS, and medical costs were analyzed using a generalized estimating equation (GEE) model with a gamma distribution and log link function to estimate relative risk (RR). The impact of TTS on 5-year mortality was assessed using a Cox proportional hazards model for survival analysis, adjusting for independent variables. Kaplan–Meier curves and log-rank tests were applied to compare survival rates by TTS before conducting survival analysis. The period up to 1 year after diagnosis was defined as the landmark, and patients who died within 1 year were excluded to avoid immortal time bias [27]. Subgroup analyses of LOS, medical costs, and 5-year mortality were conducted by income level. Interaction effects between independent variables and income level were tested, and type 3 analyses were used to confirm statistical significance at p-value <0.05. All analyses were conducted using SAS version 9.4.2 (SAS Institute Inc., Cary, NC, USA) and R version 4.0.3 (R Foundation for Statistical Computing, Vienna, Austria).

### Ethics statement

The requirements for informed consent and ethical approval were waived by the Institutional Review Board of the National Cancer Center, Korea (NCC2022-0122). The study adhered to the principles of the Declaration of Helsinki.

## RESULTS

### Patient characteristics

The general characteristics of patients with lung, liver, and colorectal cancers are presented in Table 1, Supplementary Materials 1 and 2. The mean TTS values were 15.0 days for lung cancer, 14.3 days for liver cancer, and 13.0 days for colorectal cancer. Among patients who underwent surgical treatment, 4,660 (20.4%) with lung cancer, 3,510 (11.4%) with liver cancer, and 7,001 (11.4%) with colorectal cancer experienced surgical delays of more than 30 days. The LOS for patients treated after 30 days was 112.6 days for lung cancer, 164.0 days for liver cancer, and 231.8 days for colorectal cancer, all longer than for patients treated within 30 days (p<0.001). Similarly, medical costs were higher among patients with delayed surgery: 50.3 million Korean won (KRW) for lung cancer, 78.1 million KRW for liver cancer, and 51.8 million KRW for colorectal cancer (p<0.001). Mortality within 5 years of diagnosis was 3,642 (20.0%) for lung cancer, 10,403 (38.2%) for liver cancer, and 10,035 (18.5%) for colorectal cancer among those treated within 30 days. In contrast, among patients treated after 30 days, mortality rates were significantly higher, with 1,213 (26.0%) for lung cancer, 1,563 (44.5%) for liver cancer, and 1,567 (22.4%) for colorectal cancer (p<0.001).

### Kaplan–Meier analysis

Kaplan–Meier survival curves for 5-year mortality according to TTS are presented in Supplementary Materials 3-5. The cumulative incidence of 5-year mortality was significantly higher among patients with TTS exceeding 30 days compared with those with TTS within 30 days (p<0.001). Visual inspection of the survival curves for lung, liver, and colorectal cancers indicated that the proportional hazards assumption was not violated.

### Regression analyses

Regression analyses using the GEE model and Cox proportional hazards model examined the impact of TTS on LOS, medical costs, and 5-year mortality (Table 2, Supplementary Materials 6-8). Patients with TTS exceeding 30 days had significantly higher RRs for LOS and medical costs within 5 years of diagnosis. For lung cancer, the RRs were 1.15 (95% confidence interval [CI], 1.07 to 1.24) for LOS and 1.14 (95% CI, 1.10 to 1.19) for costs. For liver cancer, the RRs were 1.26 (95% CI, 1.19 to 1.33) for LOS and 1.29 (95% CI, 1.24 to 1.34) for costs. For colorectal cancer, the RRs were 1.14 (95% CI, 1.08 to 1.20) for LOS and 1.11 (95% CI, 1.08 to 1.14) for costs. Similarly, patients with delayed surgery had higher hazard ratios for 5-year mortality: 1.15 (95% CI, 1.08 to 1.23) for lung cancer, 1.22 (95% CI, 1.16 to 1.29) for liver cancer, and 1.14 (95% CI, 1.08 to 1.21) for colorectal cancer.

### Subgroup analyses

**Figure 2.**
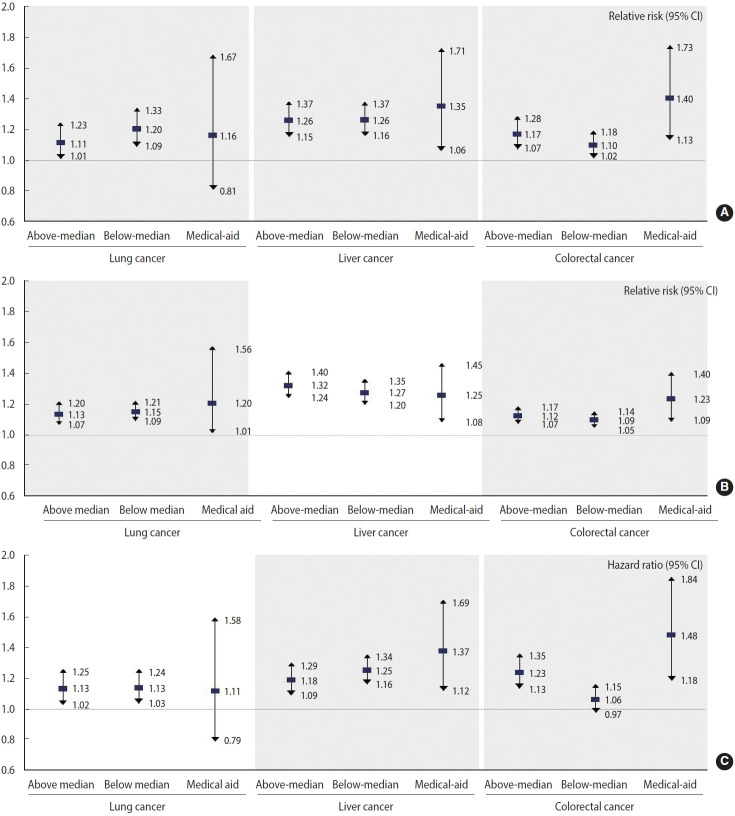
Subgroup analyses of (A) length of stay, (B) medical costs, and (C) 5-year mortality based on income level. The colored grid represents significance of interaction effect at p<0.05, for type 3 analysis. CI, confidence interval.

Subgroup analyses explored disparities in LOS, medical costs, and 5-year mortality by economic status (Figure 2, Supplementary Materials 9 and 10). Significant interaction terms were found for most comparisons, except for income levels and 5-year mortality in lung cancer (p=0.198) and income levels and medical costs in liver cancer (p=0.469).

For LOS within 5 years of diagnosis, the RRs among lung cancer patients were 1.16 (95% CI, 0.81 to 1.67) for the medical-aid group, 1.20 (95% CI, 1.09 to 1.33) for the below-median income group, and 1.11 (95% CI, 1.01 to 1.23) for the above-median income group. For liver cancer, RRs were 1.35 (95% CI, 1.06 to 1.71) for the medical-aid group, 1.26 (95% CI, 1.16 to 1.37) for the below-median group, and 1.26 (95% CI, 1.15 to 1.37) for the above-median group. For colorectal cancer, the RRs were 1.40 (95% CI, 1.13 to 1.73) for the medical-aid group, 1.10 (95% CI, 1.02 to 1.18) for the below-median group, and 1.17 (95% CI, 1.07 to 1.28) for the above-median group.

For medical costs within 5 years, the RRs among lung cancer patients were 1.20 (95% CI, 1.01 to 1.20) for the medical-aid group, 1.15 (95% CI, 1.09 to 1.21) for the below-median group, and 1.13 (95% CI, 1.07 to 1.20) for the above-median group. For liver cancer, the RRs were 1.25 (95% CI, 1.08 to 1.45) for the medical-aid group, 1.27 (95% CI, 1.20 to 1.35) for the below-median group, and 1.32 (95% CI, 1.24 to 1.40) for the above-median group. For colorectal cancer, the RRs were 1.23 (95% CI, 1.09 to 1.40) for the medical-aid group, 1.09 (95% CI, 1.05 to 1.14) for the below-median group, and 1.12 (95% CI, 1.07 to 1.17) for the above-median group.

For 5-year mortality, HRs among lung cancer patients were 1.11 (95% CI, 0.79 to 1.58) for the medical-aid group, 1.13 (95% CI, 1.03 to 1.24) for the below-median group, and 1.13 (95% CI 1.02 to 1.25) for the above-median group. For liver cancer, the HRs were 1.37 (95% CI, 1.12 to 1.69) for the medical-aid group, 1.25 (95% CI, 1.16 to 1.34) for the below-median group, and 1.18 (95% CI, 1.09 to 1.29) for the above-median group. For colorectal cancer, the HRs were 1.48 (95% CI, 1.18 to 1.84) for the medical-aid group, 1.06 (95% CI, 0.97 to 1.15) for the below-median group, and 1.23 (95% CI, 1.13 to 1.35) for the above-median group.

## DISCUSSION

### Key findings

In this study, we examined the association between delays in TTS, healthcare utilization, and mortality among patients with cancer, and we aimed to provide policy-relevant evidence. We also evaluated differences according to patient income levels. Our findings showed that 5-year mortality was significantly higher among patients who underwent surgery after 30 days than among those who received surgical treatment within 30 days. Regarding healthcare utilization, patients who experienced delays beyond 30 days had longer hospital stays and incurred higher medical costs. Furthermore, we found that lower income was associated with greater disparities in LOS, medical costs, and 5-year mortality.

### Interpretations

Compared with countries such as England, the United States, and Canada, Korea demonstrates commendable timeliness in initiating surgical treatment [21,28,29]. This achievement is likely attributable to nationwide QA programs designed to improve the overall quality of cancer care [30]. In our study, a high proportion of patients underwent surgery within 30 days of diagnosis—63.7% for lung cancer, 88.6% for liver cancer, and 88.6% for colorectal cancer. However, a subset of patients who experienced treatment delays faced longer hospital stays, greater medical expenditures, and higher mortality risks.

These associations proved robust regardless of differences in the relative 5-year survival rates of specific cancer types (lung cancer: 38.5%, liver cancer: 39.3%, colorectal cancer: 74.3% between 2017 and 2021) [31]. While previous research established an association between delayed TTS and mortality [32], our study demonstrated that delays were also associated with prolonged LOS and higher economic burden. Delays in surgery may lead to extended hospitalization for symptom management, preoperative evaluations, or administrative processes. These factors increase hospital resource consumption, reduce efficiency, and ultimately undermine the quality of care [33]. Moreover, the economic burden associated with cancer treatment plays a crucial role in treatment adherence, as financial strain is a known driver of treatment discontinuation [34]. Interruptions in cancer treatment are directly linked to poorer prognoses, and deaths resulting from such interruptions are considered avoidable, as they can often be prevented with timely and appropriate care [35].

Previous studies suggest that delays in cancer surgery are influenced by multiple, interrelated factors spanning patient, provider, and healthcare system levels [36-38]. For example, patients may voluntarily postpone surgery in pursuit of what they perceive to be higher-quality care or due to personal circumstances [36]. Systemic constraints, such as shortages in operating room capacity, referral coordination gaps, or uneven distribution of specialized personnel and resources, may also contribute [37]. In addition, patients frequently travel outside their residential regions to access care at high-volume hospitals, which are often concentrated in the capital area [38,39]. Such travel can introduce logistical barriers, scheduling bottlenecks, and increased demand at tertiary centers, thereby lengthening TTS.

Given this complexity, surgical delays cannot be attributed to centralization alone. Instead, a nuanced understanding of how patient behaviors, clinical decisions, and systemic resource limitations intersect is required. Policy strategies should therefore focus on optimizing the geographic distribution of surgical capacity, reinforcing clinical pathways between institutions, and ensuring timely access to quality care irrespective of residence. First, financial and administrative incentives should be introduced to expand surgical capacity at regional hospitals. Korea currently operates 13 regional cancer centers designed to decentralize cancer care [40]. Building upon existing initiatives, such as the Ministry of Health and Welfare’s Measures to Strengthen Regional Healthcare launched in 2019, performance-based payments for timely surgery could be scaled to encourage hospitals in non-metropolitan areas to reduce TTS and absorb more patient demand [41]. Second, TTS indicators should be incorporated into the national Healthcare Quality Assessment framework led by the HIRA. Current QA evaluations emphasize 5-year survival rates and adherence to clinical guidelines, but TTS is not systematically measured. Incorporating timeliness metrics—similar to those adopted by the United Kingdom’s National Cancer Audit Programme—could institutionalize surgical promptness as a core performance goal and promote benchmarking across hospitals [42]. Third, beyond incentivizing hospitals, policies should also guide patient choices. Strengthening regional differentiation through service specialization (e.g., by cancer type or surgical technique) and clearly communicating these roles to the public could help balance patient distribution. Public education campaigns and transparent reporting of quality outcomes would empower patients to select high-performing regional hospitals with greater confidence. These proposed strategies align with Korea’s existing healthcare governance framework while drawing on international best practices. By enhancing regional infrastructure, revising incentive structures, and promoting patient-centered service differentiation, these reforms could reduce delays, improve equity, and strengthen the overall efficiency of cancer treatment delivery nationwide.

The subgroup analysis revealed a clear trend: lower income levels were associated with greater delays in cancer treatment, resulting in prolonged LOS, higher medical costs, and increased mortality rates. These findings highlight the strong link between income disparity and delays in cancer surgery. A major challenge lies in the rational selection of healthcare services, given the information asymmetry between providers and patients. Previous research has shown that disparities in health literacy exist among income groups [43]. Low-income individuals, in particular, may struggle to choose appropriate medical institutions for cancer treatment due to limited health literacy, which hinders informed decision-making [44].

Our findings further confirm these disparities by demonstrating that the proportion of patients undergoing surgery within 30 days of diagnosis increased with income level (e.g., for colorectal cancer: 57.2% in the lowest-income group vs. 93.4% in the highest). Additionally, across all cancer types examined, patients in the lowest-income group (e.g., Medical Aid recipients) with TTS exceeding 30 days incurred the highest medical costs (63.2 million KRW for lung cancer, 66.2 million KRW for liver cancer, and 53.7 million KRW for colorectal cancer) and exhibited the highest mortality rates. These results indicate that delays in TTS disproportionately burden socioeconomically disadvantaged groups, compounding both financial strain and clinical risk.

The inefficiencies in healthcare resource use, the elevated medical expenditures, and the poorer outcomes associated with delays in TTS emphasize the urgent need to secure timely surgical intervention for cancer patients, particularly those in lower-income groups. To achieve this, it is critical to enhance the competitiveness of regional hospitals by fostering specialized clinical services and strengthening their capacity for advanced treatments. The Japanese Clinical Oncology Group provides a valuable example, having established a tiered national network that integrates clinical trial activity, specialized services, and advanced technologies across hospitals of varying levels [45]. This approach facilitates seamless care pathways between institutions and ensures high-quality, timely cancer care regardless of patient location [46,47]. Developing a similarly competitive and well-coordinated cancer care network in Korea would help alleviate the overconcentration of patients in major tertiary hospitals, reduce treatment delays, and improve equity in cancer care delivery nationwide. The benefits would be particularly significant for socioeconomically disadvantaged populations, who often face logistical, financial, and informational barriers to receiving timely care.

### Limitations

This study has several limitations. First, it did not include health behavior variables such as alcohol consumption, smoking, and physical activity, which may influence health outcomes. To mitigate this, we incorporated the CCI to reflect participants’ underlying health status. Second, patients who died within 1-year of diagnosis—likely representing more severe cases—were excluded. Third, discrepancies may exist between actual diagnostic information and claimed codes. However, in Korea, a policy ensures that 95% of medical costs for confirmed cancer patients are covered under the specific cancer code “V193.” This reduces the likelihood of substantial coding mismatches in our dataset. Fourth, the study used medical costs as an outcome variable, which could require standardization for currency value changes over time. To address this, we included the year of diagnosis as a covariate in the models to adjust for expenditure trends (Supplementary Materials 6-8). Fifth, the study relied on NHIS administrative claims data, which lack detailed clinical information such as surgical appropriateness, provider expertise, and other qualitative aspects of cancer care. These factors may influence treatment quality, but could not be evaluated here. Future studies should incorporate patient-level and provider-level perspectives to allow for a more comprehensive assessment of surgical appropriateness and clinical expertise. Sixth, the dataset did not allow for detailed examination of the causes of surgical delay, especially in determining whether observed disparities among lower-income groups stemmed from delays in surgery versus delays in diagnosis. Claims data only capture information from the point of diagnosis, excluding clinical details such as stage at presentation, diagnostic pathways, or symptom onset. These variables are critical for evaluating diagnostic delay. Future research incorporating clinical and diagnostic data is needed to clarify how patient-level and system-level barriers interact in driving surgical delays. Finally, cancer staging information was not available in the claims database, limiting our ability to directly account for disease severity. Although we adjusted for comorbidity burden using the CCI, and incorporated treatment types (e.g., chemotherapy, radiotherapy) as proxies, these may not fully reflect clinical heterogeneity. Evidence suggests that socioeconomic status may influence treatment delay and outcomes differently by stage, with disparities more pronounced in early-stage disease but less evident in advanced-stage cases where clinical urgency predominates [48,49]. We could not explore this interaction due to the absence of staging data. To partially address this limitation, we excluded patients who died within 1 year of diagnosis or who did not receive treatment during the first year, as such cases may reflect advanced or untreatable disease. Despite these limitations, this study provides meaningful evidence on the association between delayed TTS, increased healthcare utilization, and higher mortality among cancer patients. It also underscores the significant role of income disparities in shaping treatment timeliness and outcomes.

These findings highlight the urgent need to improve the efficiency of Korea’s healthcare delivery system and to reduce health literacy gaps that hinder patient decision-making regarding treatment institutions. Addressing these issues will be critical to minimizing treatment delays, improving equity, and ultimately enhancing cancer care outcomes.

## Figures and Tables

**Figure f3-epih-47-e2025056:**
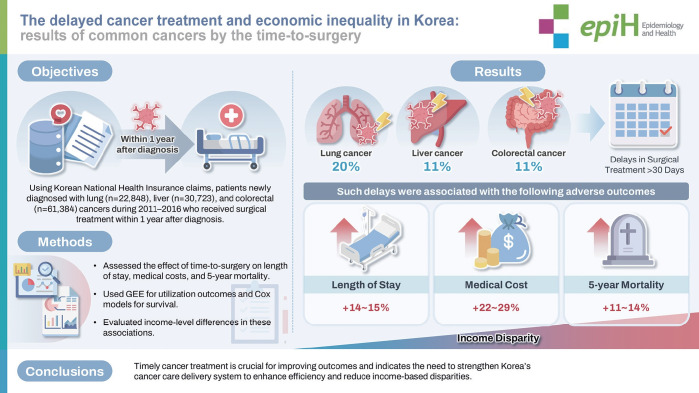


**Table 1.** Associations between TTS, LOS, medical costs, and 5-year mortality among patients with lung, liver, and colorectal cancer

Type of cancer	TTS (day)	Total^1^	LOS (day)	p-value	Medical costs (10^6^ KRW)	p-value	Mortality		
							Survived	Died	p-value
Lung cancer		15.0±20.4	-		-		-	-	
	Median	11.0	-		-		-	-	
	≤30	63.7	86.9±181.4	<0.001	38.4±47.7	<0.001	14,546 (80.0)	3,642 (20.0)	<0.001
	>30	36.3	112.6±211.8		50.3±62.2		3,447 (74.0)	1,213 (26.0)	
Liver cancer^1^		14.3±30.1	-		-		-	-	
	Median	6.0	-		-		-	-	
	≤30	88.6	131.6±216.0	<0.001	61.4±73.2	<0.001	16,810 (61.8)	10,403 (38.2)	<0.001
	>30	11.4	164.0±239.5		78.1±97.7		1,947 (55.5)	1,563 (44.5)	
Colorectal cancer^1^		13.0±21.01	-		-		-	-	
	Median	5.0	-		-		-	-	
	≤30	88.6	107.8±138.2	<0.001	36.8±48.1	<0.001	44,348 (81.5)	10,035 (18.5)	<0.001
	>30	11.4	231.8±253.9		51.8±56.7		5,434 (77.6)	1,567 (22.4)	

Values are presented as mean±standard deviation or number (%). TTS, time-to-surgery; LOS, length of stay; SD, standard deviation; KRW, Korean won.

1 Number of total participants based on experience of delay in surgical treatment.

**Table 2.** The results of regression analyses^1^ and survival analysis for investigating the relationships between TTS, medical LOS, medical costs, and 5-year mortality based on the type of cancer

Type of cancer	TTS (day)	LOS	Medical costs	Mortality
		RR (95% CI)	RR (95% CI)	HR (95% CI)
Lung cancer	≤30	1.00 (reference)	1.00 (reference)	1.00 (reference)
	>30	1.15 (1.07, 1.24)	1.14 (1.10, 1.19)	1.15 (1.08, 1.23)
Liver cancer	≤30	1.00 (reference)	1.00 (reference)	1.00 (reference)
	>30	1.26 (1.19, 1.33)	1.29 (1.24, 1.34)	1.22 (1.16, 1.29)
Colorectal cancer	≤30	1.00 (reference)	1.00 (reference)	1.00 (reference)
	>30	1.14 (1.08, 1.20)	1.11 (1.08, 1.14)	1.14 (1.08, 1.21)

TTS, time-to-surgery; LOS, length of stay; RR, relative risk; HR, hazard ratio; CI, confidence interval.

1 Regression analyses using a generalized estimating equation model for LOS, and medical costs, and survival analysis using a Cox proportional hazard model for 5-year mortality after controlling for covariates.

## Supplementary materials

Supplementary Material 1. Characteristics of the study population based on medical costs and LOS

Supplementary Material 2. Characteristics of the study population based on 5-year mortality

Supplementary Material 3. Kaplan–Meier survival curves and results of Log-rank test for five-year mortality for lung cancer

Supplementary Material 4. Kaplan–Meier survival curves and results of Log-rank test for five-year mortality for liver cancer

Supplementary Material 5. Kaplan–Meier survival curves and results of Log-rank test for five-year mortality for colorectal cancer

Supplementary Material 6. The result of regression analysis using GEE model investigating relationship between TTS and LOS

Supplementary Material 7. The result of regression analysis using GEE model investigating relationship between TTS and medical costs

Supplementary Material 8. The result of survival analysis using Cox proportional hazard model investigating relationship between TTS and 5-year mortality

Supplementary Material 9. Characteristics of the study population based on income level and 5-year mortality

Supplementary Material 10. Characteristics of the study population based on income level, LOS, and Medical costs
