# Supplementary material for: The delayed cancer treatment and economic inequality in Korea: results of common cancers by the time-to-surgery
Source: Epidemiol Health. 2025 Sep 27;47:e2025056. doi: 10.4178/epih.e2025056 (PMC12869139; doi:10.4178/epih.e2025056)
Supplement: Supplementary Material 1. — Characteristics of the study population based on medical costs and LOS [file epih-47-e2025056-Supplementary-1.docx]

Supplementary Material 1. Characteristics of the study population based on medical costs and LOS

| **Variable** | **LOS (Unit: days)** | | | | | | **Medical costs (Unit: 1,000,000￦)** | | | | | |
| --- | --- | --- | --- | --- | --- | --- | --- | --- | --- | --- | --- | --- |
|  | **Lung cancer** | | **Liver cancer** | | **Colorectal cancer** | | **Lung cancer** | | **Liver cancer** | | **Colorectal cancer** | |
|  | **Mean (±SD)** | **p-value** | **Mean (±SD)** | **p-value** | **Mean (±SD)** | **p-value** | **Mean (±SD)** | **p-value** | **Mean (±SD)** | **p-value** | **Mean (±SD)** | **p-value** |
| **Total** | 92.1  (187.3) | **-** | 135.3 (218.0) | **-** | 111.2 (231.9) | **-** | 40.8 (51.0) | **-** | 63.3 (76.2) | **-** | 38.5 (48.9) |  |
| **TTS** | | | | | | | | | | | | |
| ≤30 days | 86.9 (181.4) | <.001 | 131.6 (216.0) | <.001 | 107.8 (231.8) | <.001 | 38.4 (47.7) | <.001 | 61.4 (73.2) | <.001 | 36.8 (48.1) | <.001 |
| >30 days | 112.6 (211.8) |  | 164.0 (239.5) |  | 138.2 (253.9) |  | 50.3 (62.2) |  | 78.1 (97.7) |  | 51.8 (56.7) |  |
| **Gender** | | | | | | | | | | | | |
| Men | 102.6 (194.8) | <.001 | 133.3 (213.8) | 0.005 | 104.8 (216.8) | <.001 | 46.7 (57.1) | <.001 | 65.2 (79.7) | <.001 | 39.9 (50.8) | <.001 |
| Women | 76.7 (177.1) |  | 142.3 (236.4) |  | 119.8 (256.0) |  | 32.1 (39.4) |  | 56.7 (64.3) |  | 36.7 (47.5) |  |
| **Age (years)** | | | | | | | | | | | | |
| ≤54 | 91.9 (202.3) | 0.001 | 136.5 (231.2) | <.001 | 99.6 (256.0) | <.001 | 38.8 (50.3) | 0.001 | 70.7 (89.5) | <.001 | 40.4 (54.1) | <.001 |
| 55-64 | 88.8 (183.3) |  | 126.2 (198.0) |  | 95.7 (192.9) |  | 40.5 (52.5) |  | 62.8 (77.5) |  | 39.5 (50.1) |  |
| 65-74 | 91.6 (177) |  | 135.2 (211.1) |  | 103.4 (215.6) |  | 42.5 (51.1) |  | 55.6 (57.2) |  | 37.4 (46.9) |  |
| ≥75 | 105.9 (210) |  | 171.1 (264.8) |  | 162.7 (339.6) |  | 40.5 (48.5) |  | 52.2 (44.7) |  | 36.1 (44.8) |  |
| **Income level** | | | | | | | | | | | | |
| Medical-aid | 151.2 (266.9) | <.001 | 195.0 (287.3) | <.001 | 204.2 (370.6) | <.001 | 44.4 (51.6) | <.001 | 58.7 (56.9) | <.001 | 42.4 (49.9) | <.001 |
| Below median | 97.5 (193.9) |  | 141.2 (231.4) |  | 112.2 (226.9) |  | 42.7 (52.7) |  | 64.5 (80.6) |  | 40.3 (50.9) |  |
| Above median | 82.1 (174.0) |  | 123.1 (195.6) |  | 101.5 (224.4) |  | 38.5 (49.4) |  | 62.6 (74.3) |  | 36.2 (47.5) |  |
| **Residing area** | | | | | | | | | | | | |
| Seoul | 71.3 (156.1) | <.001 | 122.2 (199.9) | <.001 | 90.7 (195.3) | <.001 | 37.6 (51.3) | <.001 | 62.7 (81.8) | <.001 | 37.6 (49.1) | 0.025 |
| Other metropolitan | 105.2 (206.8) |  | 146.9 (228.4) |  | 126.8 (261.4) |  | 42.2 (52.0) |  | 63.8 (73.5) |  | 39.4 (50.7) |  |
| Non-metropolitan | 94.5 (190.6) |  | 134.5 (220.7) |  | 111.9 (234.7) |  | 41.5 (50.7) |  | 63.3 (76.2) |  | 38.4 (48.9) |  |
| **Type of treatment** | | | | | | | | | | | | |
| Only surgery | 58.1 (144.7) | <.001 | 87.7 (179.6) | <.001 | 102.7 (235.0) | <.001 | 26.5 (33.5) | <.001 | 45.4 (68.8) | <.001 | 32.5 (43.5) | <.001 |
| Surgery with chemotherapy or radiotherapy | 156.4 (238.0) |  | 180.5 (242.4) |  | 141.5 (230.6) |  | 68.0 (65.8) |  | 80.3 (79.7) |  | 59.7 (61.7) |  |
| **Type of major treatment institution** | | | | | | | | | | | | |
| Tertiary | 87.4 (183.3) | <.001 | 131.3 (208.3) | <.001 | 103.4 (220.1) | <.001 | 39.7 (50.5) | <.001 | 63.9 (75.9) | 0.009 | 39.3 (50.6) | <.001 |
| others | 108.7 (204.1) |  | 149.7 (253.6) |  | 124.6 (257.0) |  | 44.6 (53.3) |  | 61.1 (79.1) |  | 37.1 (47.3) |  |
| **Multiple cancer** | | | | | | | | | | | | |
| No | 56.9 (155.5) | <.001 | 101.8 (201.8) | <.001 | 72.5 (215.0) | <.001 | 25.6 (36.9) | <.001 | 49.0 (67.0) | <.001 | 21.5 (28.3) | <.001 |
| Yes | 133.9 (213.6) |  | 190.8 (234.8) |  | 151.4 (247.0) |  | 58.9 (59.2) |  | 86.9 (85.2) |  | 56.2 (59.4) |  |
| p<0.001^***^ , p<0.01^**^, p<0.05^*^  Abbreviation: TTS: Time to surgery; LOS: Length of stay; CCI:Charlson comorbidity index | | | | | | | | | | | | |
