# Supplementary material for: The delayed cancer treatment and economic inequality in Korea: results of common cancers by the time-to-surgery
Source: Epidemiol Health. 2025 Sep 27;47:e2025056. doi: 10.4178/epih.e2025056 (PMC12869139; doi:10.4178/epih.e2025056)
Supplement: Supplementary Material 2. — Characteristics of the study population based on 5-year mortality [file epih-47-e2025056-Supplementary-2.docx]

| **Supplementary Material** **2. Characteristics of the study population based on 5-year mortality** | | | | | | | | | | | | | | | | | | |
| --- | --- | --- | --- | --- | --- | --- | --- | --- | --- | --- | --- | --- | --- | --- | --- | --- | --- | --- |
| **Variable** | **5-year mortality** | | | | | | | | | | | | | | | | | |
|  | **Lung cancer** | | | | | | **Liver cancer** | | | | | | **Colorectal cancer** | | | | | |
|  | **Total** | **Survived** | | **Died** | | **p-value** | **Total** | **Survived** | | **Died** | | **p-value** | **Total** | **Survived** | | **Died** | | **p-value** |
|  |  | **N/**  **Mean** | **%/**  **SD** | **N/**  **Mean** | **%/**  **SD** |  |  | **N/**  **Mean** | **%/**  **SD** | **N/**  **Mean** | **%/**  **SD** |  |  | **N/**  **Mean** | **%/**  **SD** | **N/Mean** | **%/SD** |  |
| **Total** | 22848 | 17993 | 78.8 | 4855 | 21.3 | - | 30723 | 18757 | 61.1 | 11966 | 39.0 | - | 61384 | 49782 | 81.1 | 11602 | 18.9 | - |
| **TTS** | | | | | | | | | | | | | |  |  |  |  |  |
| ≤30 days | 18188 | 14546 | 80.0 | 3642 | 20.0 | <.001 | 27213 | 16810 | 61.8 | 10403 | 38.2 | <.001 | 54383 | 44348 | 81.5 | 10035 | 18.5 | <.001 |
| >30 days | 4660 | 3447 | 74.0 | 1213 | 26.0 |  | 3510 | 1947 | 55.5 | 1563 | 44.5 |  | 7001 | 5434 | 77.6 | 1567 | 22.4 |  |
| **Gender** | | | | | | | | | | | | | |  |  |  |  |  |
| Men | 13631 | 9977 | 73.2 | 3654 | 6.8 | <.001 | 23823 | 14366 | 60.3 | 9457 | 39.7 | <.001 | 34983 | 28038 | 80.1 | 6945 | 19.9 | <.001 |
| Women | 9217 | 8016 | 87.0 | 1201 | 13.0 |  | 6900 | 4391 | 63.6 | 2509 | 36.4 |  | 26401 | 21744 | 82.4 | 4657 | 17.6 |  |
| **Age (years)** | | | | | | | | | | | | | |  |  |  |  |  |
| ≤54 | 4967 | 4269 | 85.9 | 698 | 14.1 | <.001 | 11197 | 7529 | 67.2 | 3668 | 32.8 | <.001 | 16326 | 14292 | 87.5 | 2034 | 12.5 | <.001 |
| 55-64 | 7888 | 6534 | 82.8 | 1354 | 17.2 |  | 10383 | 6665 | 64.2 | 3718 | 35.8 |  | 16576 | 14252 | 86.0 | 2324 | 14.0 |  |
| 65-74 | 7752 | 5740 | 74.0 | 2012 | 26.0 |  | 6876 | 3737 | 54.3 | 3139 | 45.7 |  | 17161 | 13809 | 80.5 | 3352 | 19.5 |  |
| ≥75 | 2241 | 1450 | 64.7 | 791 | 35.3 |  | 2267 | 826 | 36.4 | 1441 | 63.6 |  | 11321 | 7429 | 65.6 | 3892 | 34.4 |  |
| **Income level** | | | | | | | | | | | | | |  |  |  |  |  |
| Medical-aid | 753 | 538 | 71.4 | 215 | 28.6 | <.001 | 1515 | 697 | 46.0 | 818 | 54.0 | <.001 | 2613 | 1835 | 70.2 | 778 | 29.8 | <.001 |
| Below median | 11450 | 8998 | 78.6 | 2452 | 21.4 |  | 14680 | 8882 | 60.5 | 5798 | 39.5 |  | 30893 | 25141 | 81.4 | 5752 | 18.6 |  |
| Above median | 10645 | 8457 | 79.4 | 2188 | 20.6 |  | 14528 | 9178 | 63.2 | 5350 | 36.8 |  | 27878 | 22806 | 81.8 | 5072 | 18.2 |  |
| **Residing area** | | | | | | | | | | | | | |  |  |  |  |  |
| Seoul | 4903 | 4021 | 82.0 | 882 | 18.0 | <.001 | 5872 | 3730 | 63.5 | 2142 | 36.5 | <.001 | 12566 | 10459 | 83.2 | 2107 | 16.8 | <.001 |
| Other metropolitan | 5550 | 4352 | 78.4 | 1198 | 21.6 |  | 7852 | 4686 | 59.7 | 3166 | 40.3 |  | 15136 | 12291 | 81.2 | 2845 | 18.8 |  |
| Non-metropolitan | 12395 | 9620 | 77.6 | 2775 | 22.4 |  | 16999 | 10341 | 60.8 | 6658 | 39.2 |  | 33682 | 27032 | 80.3 | 6650 | 19.7 |  |
| **CCI Score** | 1.7 | 1.6 | 1.5 | 1.9 | 1.6 | <.001 | 3.01 | 2.9 | 1.6 | 3.3 | 2.0 | <.001 | 1.3 | 1.2 | 1.4 | 1.6 | 1.7 | <.001 |
| **Type of treatment** | | | | | | | | | | | | | |  |  |  |  |  |
| Only surgery | 14951 | 12915 | 86.4 | 2036 | 13.6 | <.001 | 14957 | 11664 | 78.0 | 3293 | 22.0 | <.001 | 47894 | 39300 | 82.1 | 8594 | 17.9 | <.001 |
| Surgery with chemotherapy or radiotherapy | 7897 | 5078 | 64.3 | 2819 | 35.7 |  | 15766 | 7093 | 45.0 | 8673 | 55.0 |  | 13490 | 10482 | 77.7 | 3008 | 22.3 |  |
| **Type of major treatment institution** | | | | | | | | | | | | | |  |  |  |  |  |
| Tertiary | 17755 | 14195 | 79.9 | 3560 | 20.1 | <.001 | 24018 | 14996 | 62.4 | 9022 | 37.6 | <.001 | 38744 | 31879 | 82.3 | 6865 | 17.7 | <.001 |
| Others | 5093 | 3798 | 74.6 | 1295 | 25.4 |  | 6705 | 3761 | 56.1 | 2944 | 43.9 |  | 22640 | 17903 | 79.1 | 4737 | 20.9 |  |
| **Year of diagnosis** | 2014 | 2014 | 1.7 | 2014 | 1.7 | <.001 | 2014 | 2014 | 1.7 | 2013 | 1.7 | <.001 | 2013 | 2013 | 1.7 | 2014 | 1.7 | <.001 |
| **Multiple cancer** | | | | | | | | | | | | | | | | | | |
| No | 12393 | 11176 | 90.2 | 1217 | 9.8 | <.001 | 19147 | 13697 | 71.5 | 5450 | 28.5 | <.001 | 31276 | 28826 | 92.2 | 2450 | 7.8 | <.001 |
| Yes | 10455 | 6817 | 65.2 | 3638 | 34.8 |  | 11576 | 5060 | 43.7 | 6516 | 56.3 |  | 30108 | 20956 | 69.6 | 9152 | 30.4 |  |
| p<0.001^***^ , p<0.01^**^, p<0.05^*^  Abbreviation: TTS: Time to surgery; LOS: Length of stay; CCI:Charlson comorbidity index | | | | | | | | | | | | | | | | | | |
