# Supplementary material for: The delayed cancer treatment and economic inequality in Korea: results of common cancers by the time-to-surgery
Source: Epidemiol Health. 2025 Sep 27;47:e2025056. doi: 10.4178/epih.e2025056 (PMC12869139; doi:10.4178/epih.e2025056)

**Supplementary Material 5. Kaplan–Meier survival curves and results of Log-rank test for five-year mortality for colorectal cancer**


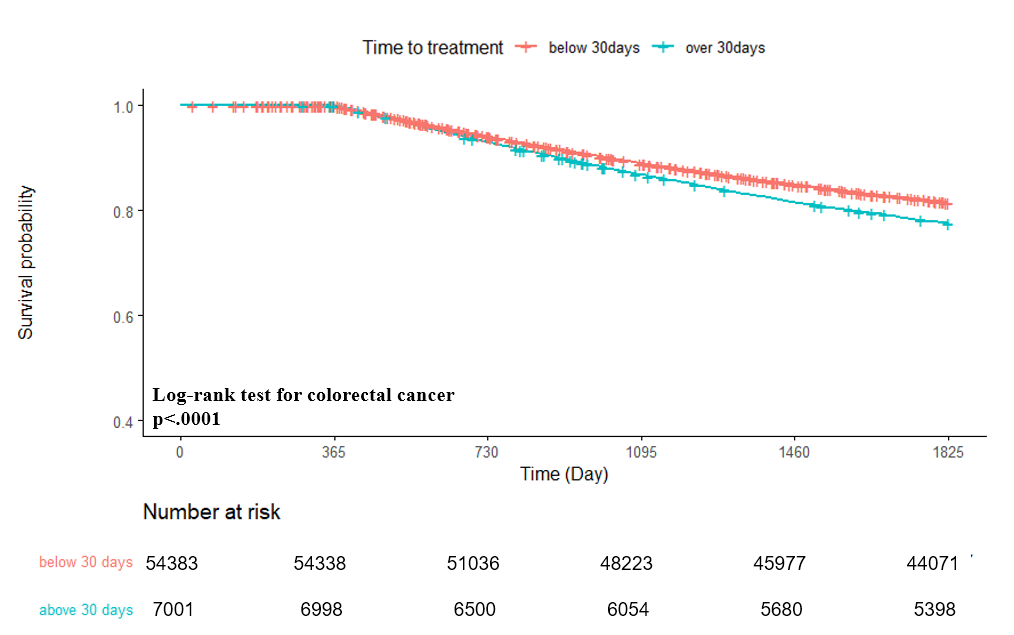

Supplement: Supplementary Material 5. — Kaplan–Meier survival curves and results of Log-rank test for five-year mortality for colorectal cancer [file epih-47-e2025056-Supplementary-5.docx]
