# Supplementary material for: The delayed cancer treatment and economic inequality in Korea: results of common cancers by the time-to-surgery
Source: Epidemiol Health. 2025 Sep 27;47:e2025056. doi: 10.4178/epih.e2025056 (PMC12869139; doi:10.4178/epih.e2025056)
Supplement: Supplementary Material 6. — The result of regression analysis using GEE model investigating relationship between TTS and LOS [file epih-47-e2025056-Supplementary-6.docx]

Supplementary Material 6. The result of regression analysis using GEE model investigating relationship between TTS and LOS

| **Variable** | **LOS** | | | | | |
| --- | --- | --- | --- | --- | --- | --- |
|  | **Lung cancer** | | **Liver cancer** | | **Colorectal cancer** | |
|  | **RR** | **95% CI** | **RR** | **95% CI** | **RR** | **95% CI** |
| **TTS** | | | | | | |
| ≤30 days | 1.00 |  | 1.00 |  | 1.00 |  |
| >30 days | 1.15 | (1.07–1.24) | 1.26 | (1.19–1.33) | 1.14 | (1.08–1.20) |
| **Gender** | | | | | | |
| Men | 1.00 |  | 1.00 |  | 1.00 |  |
| Women | 0.89 | (0.84–0.95) | 1.06 | (1.01–1.11) | 1.08 | (1.05–1.12) |
| **Age (years)** | | | | | | |
| ≤54 | 1.00 |  | 1.00 |  | 1.00 |  |
| 55-64 | 0.97 | (0.89–1.05) | 0.92 | (0.88–0.96) | 0.95 | (0.91–0.99) |
| 65-74 | 1.10 | (1.02–1.19) | 1.04 | (0.99–1.10) | 1.03 | (0.98–1.08) |
| ≥75 | 1.74 | (1.54–1.96) | 1.35 | (1.24–1.47) | 1.85 | (1.75–1.96) |
| **Income level** | | | | | | |
| Medical-aid | 1.00 |  | 1.00 |  | 1.00 |  |
| Below median | 0.59 | (0.49–0.70) | 0.88 | (0.84–0.93) | 0.62 | (0.56–0.67) |
| Above median | 0.50 | (0.41–0.60) | 0.79 | (0.75–0.82) | 0.53 | (0.49–0.58) |
| **Residing area** | | | | | | |
| Seoul | 1.00 |  | 1.00 |  | 1.00 |  |
| Other metropolitan | 1.46 | (1.34–1.60) | 1.18 | (1.11–1.25) | 1.36 | (1.29–1.43) |
| Non-metropolitan | 1.28 | (1.18–1.38) | 1.11 | (1.05–1.16) | 1.17 | (1.12–1.23) |
| **CCI score** | 1.10 | (1.08–1.12) | 1.09 | (1.08–1.11) | 1.12 | (1.11–1.14) |
| **Type of treatment** | | | | | | |
| Only surgery | 1.00 |  | 1.00 |  | 1.00 |  |
| Surgery with chemotherapy or radiotherapy | 2.44 | (2.30–2.58) | 2.06 | (1.98–2.15) | 1.41 | (1.35–1.47) |
| **Type of major treatment institution** | | | | | | |
| Tertiary | 1.00 |  | 1.00 |  | 1.00 |  |
| Others | 1.15 | (1.07–1.24) | 1.08 | (1.02–1.13) | 1.12 | (1.08–1.16) |
| **Year of diagnosis** | 0.98 | (0.96–0.99) | 1.03 | (1.02–1.04) | 1.02 | (1.01–1.03) |
| **Multiple cancer** | | | | | | |
| No | 1.00 |  | 1.00 |  | 1.00 |  |
| Yes | 2.01 | (1.89–2.14) | 1.94 | (1.87–2.01) | 2.36 | (2.28–2.45) |
| A regression analysis using GEE model with gamma distribution and log-link function  Abbreviation: TTS: Time to surgery; LOS: Length of stay; RR: Relative risk; CI:confidence interval; CCI:Charlson comorbidity index | | | | | | |
