# Supplementary material for: The delayed cancer treatment and economic inequality in Korea: results of common cancers by the time-to-surgery
Source: Epidemiol Health. 2025 Sep 27;47:e2025056. doi: 10.4178/epih.e2025056 (PMC12869139; doi:10.4178/epih.e2025056)
Supplement: Supplementary Material 7. — The result of regression analysis using GEE model investigating relationship between TTS and medical costs [file epih-47-e2025056-Supplementary-7.docx]

Supplementary Material 7. The result of regression analysis using GEE model investigating relationship between TTS and medical costs

| **Variable** | **Medical costs** | | | | | |
| --- | --- | --- | --- | --- | --- | --- |
|  | **Lung cancer** | | **Liver cancer** | | **Colorectal cancer** | |
|  | **RR** | **95% CI** | **RR** | **95% CI** | **RR** | **95% CI** |
| **TTS** | | | | | | |
| ≤30 days | 1.00 |  | 1.00 |  | 1.00 |  |
| >30 days | 1.14 | (1.10–1.19) | 1.29 | (1.24–1.34) | 1.11 | (1.08–1.14) |
| **Gender** |  |  |  |  |  |  |
| Men | 1.00 |  | 1.00 |  | 1.00 |  |
| Women | 0.81 | (0.78–0.83) | 0.95 | (0.92–0.98) | 0.96 | (0.94–0.98) |
| **Age (years)** | | | | | | |
| ≤54 | 1.00 |  | 1.00 |  | 1.00 |  |
| 55-64 | 1.03 | (0.99–1.06) | 0.87 | (0.84–0.90) | 0.98 | (0.96–1.01) |
| 65-74 | 1.16 | (1.12–1.21) | 0.78 | (0.75–0.81) | 0.98 | (0.96–1.00) |
| ≥75 | 1.36 | (1.28–1.45) | 0.74 | (0.70–0.77) | 1.09 | (1.06–1.12) |
| **Income level** | | | | | | |
| Medical-aid | 1.00 |  | 1.00 |  | 1.00 |  |
| Below median | 0.90 | (0.81–1.00) | 0.99 | (0.96–1.03) | 0.91 | (0.86–0.95) |
| Above median | 0.84 | (0.76–0.94) | 1.01 | (0.98–1.05) | 0.85 | (0.81–0.89) |
| **Residing area** | | | | | | |
| Seoul | 1.00 |  | 1.00 |  | 1.00 |  |
| Other metropolitan | 1.07 | (1.02–1.12) | 0.98 | (0.95–1.02) | 1.02 | (1.00–1.05) |
| Non-metropolitan | 1.03 | (0.99–1.07) | 1.00 | (0.97–1.04) | 1.01 | (0.99–1.04) |
| **CCI Score** | 1.04 | (1.03–1.05) | 1.06 | (1.05–1.08) | 1.05 | (1.04–1.06) |
| **Type of treatment** | | | | | | |
| Only surgery | 1.00 |  | 1.00 |  | 1.00 |  |
| Surgery with chemotherapy or radiotherapy | 2.17 | (2.11–2.24) | 1.83 | (1.77–1.88) | 1.55 | (1.51–1.58) |
| **Type of major treatment institution** | | | | | | |
| Tertiary | 1.00 |  | 1.00 |  | 1.00 |  |
| Others | 1.07 | (1.04–1.11) | 0.94 | (0.91–0.98) | 0.94 | (0.92–0.96) |
| **Year of diagnosis** | 1.04 | (1.03–1.05) | 1.08 | (1.07–1.09) | 1.06 | (1.05–1.06) |
| **Multiple cancer** | | | | | | |
| No | 1.00 |  | 1.00 |  | 1.00 |  |
| Yes | 1.88 | (1.83–1.94) | 1.72 | (1.67–1.76) | 2.54 | (2.49–2.58) |
| A regression analysis using GEE model with gamma distribution and log-link function  Abbreviation: TTS: Time to surgery; LOS: Length of stay; RR: Relative risk; CI:confidence interval; CCI:Charlson comorbidity index | | | | | | |
