# Supplementary material for: The delayed cancer treatment and economic inequality in Korea: results of common cancers by the time-to-surgery
Source: Epidemiol Health. 2025 Sep 27;47:e2025056. doi: 10.4178/epih.e2025056 (PMC12869139; doi:10.4178/epih.e2025056)
Supplement: Supplementary Material 8. — The result of survival analysis using Cox proportional hazard model investigating relationship between TTS and 5-year mortality [file epih-47-e2025056-Supplementary-8.docx]

Supplementary Material 8. The result of survival analysis using Cox proportional hazard model investigating relationship between TTS and 5-year mortality

| **Variable** | **5-year mortality** | | | | | |
| --- | --- | --- | --- | --- | --- | --- |
|  | **Lung cancer** | | **Liver cancer** | | **Colorectal cancer** | |
|  | **HR** | **95% CI** | **HR** | **95% CI** | **HR** | **95% CI** |
| **TTS** |  |  |  |  |  |  |
| ≤30 days | 1.00 |  | 1.00 |  | 1.00 |  |
| >30 days | 1.15 | (1.08–1.23) | 1.22 | (1.16–1.29) | 1.14 | (1.08–1.21) |
| **Gender** |  |  |  |  |  |  |
| Men | 1.00 |  | 1.00 |  | 1.00 |  |
| Women | 0.57 | (0.53–0.61) | 0.88 | (0.84–0.92) | 0.84 | (0.80–0.87) |
| **Age (years)** |  |  |  |  |  |  |
| ≤54 | 1.00 |  | 1.00 |  | 1.00 |  |
| 55-64 | 1.13 | (1.03–1.24) | 1.06 | (1.02–1.11) | 1.06 | (0.99–1.12) |
| 65-74 | 1.79 | (1.64–1.95) | 1.41 | (1.35–1.48) | 1.51 | (1.43–1.60) |
| ≥75 | 3.18 | (2.86–3.54) | 2.27 | (2.13–2.41) | 3.15 | (2.98–3.34) |
| **Income level** |  |  |  |  |  |  |
| Medical-aid | 1.00 |  | 1.00 |  | 1.00 |  |
| Below median | 0.90 | (0.78–1.03) | 0.95 | (0.90–0.99) | 0.79 | (0.73–0.85) |
| Above median | 0.88 | (0.76–1.01) | 0.83 | (0.79–0.86) | 0.69 | (0.64–0.75) |
| **Residing area** |  |  |  |  |  |  |
| Seoul | 1.00 |  | 1.00 |  | 1.00 |  |
| Other metropolitan | 1.12 | (1.03–1.23) | 1.11 | (1.05–1.17) | 1.10 | (1.04–1.17) |
| Non-metropolitan | 1.11 | (1.03–1.20) | 1.06 | (1.01–1.11) | 1.12 | (1.06–1.17) |
| **CCI Score** | 1.05 | (1.04–1.07) | 1.08 | (1.07–1.09) | 1.08 | (1.07–1.09) |
| **Type of treatment** |  |  |  |  |  |  |
| Only surgery | 1.00 |  | 1.00 |  | 1.00 |  |
| Surgery with chemotherapy or radiotherapy | 1.13 | (1.05–1.20) | 1.04 | (1.00–1.09) | 1.02 | (0.98–1.06) |
| **Type of major treatment institution** | | | | |  |  |
| Tertiary | 1.00 |  | 1.00 |  | 1.00 |  |
| Others | 2.49 | (2.34–2.64) | 3.00 | (2.88–3.12) | 1.19 | (1.13–1.24) |
| **Year of diagnosis** | 0.94 | (0.93–0.96) | 1.01 | (1.01–1.02) | 0.98 | (0.97–0.99) |
| **Multiple cancer** |  |  |  |  |  |  |
| No | 1.00 |  | 1.00 |  | 1.00 |  |
| Yes | 3.19 | (2.98–3.40) | 2.17 | (2.10–2.25) | 4.37 | (4.18–4.57) |
| Note: NHI: National Health Insurance; HR: Hazard ratio; CI: confidence interval; CCI: Charlson comorbidity index | | | | | | |
