# Supplementary material for: The delayed cancer treatment and economic inequality in Korea: results of common cancers by the time-to-surgery
Source: Epidemiol Health. 2025 Sep 27;47:e2025056. doi: 10.4178/epih.e2025056 (PMC12869139; doi:10.4178/epih.e2025056)
Supplement: Supplementary Material 9. — Characteristics of the study population based on income level and 5-year mortality [file epih-47-e2025056-Supplementary-9.docx]

| **Supplementary Material 9. Characteristics of the study population based on income level and 5-year mortality** | | | | | | | | | | | | | | | | | | | |
| --- | --- | --- | --- | --- | --- | --- | --- | --- | --- | --- | --- | --- | --- | --- | --- | --- | --- | --- | --- |
| **Variable** | **5-year mortality** | | | | | | | | | | | | | | | | | | |
|  | **Lung cancer** | | | | | | **Liver cancer** | | | | | | **Colorectal cancer** | | | | | | |
|  | **Total** | **Survived** | | **Died** | | **p-**  **value** | **Total** | **Survived** | | **Died** | | **p-**  **value** | **Total** | **Survived** | | **Died** | | **p-**  **value** |  |
|  |  | **N/Mean** | **%/SD** | **N/Mean** | **%/SD** |  |  | **N/Mean** | **%/SD** | **N/Mean** | **%/SD** |  |  | **N/Mean** | **%/SD** | **N/Mean** | **%/SD** |  |  |
| **Medical aid** | | | | | | | | | | | | | | | | | | | |
| **Total** | 753 | 538 | 71.4 | 215 | 28.6 | - | 1515 | 697 | 46.0 | 818 | 54.0 | - | 2613 | 1835 | 70.2 | 778 | 29.8 |  |  |
| **TTS** |  |  |  |  |  |  |  |  |  |  |  |  |  |  |  |  |  |  |  |
| ≤30 days | 566 | 406 | 71.4 | 160 | 28.3 | 0.04 | 1110 | 560 | 50.5 | 550 | 49.5 | <.001 | 1350 | 1050 | 77.8 | 300 | 22.2 | <.001 |  |
| >30 days | 187 | 132 | 72.1 | 55 | 29.4 |  | 405 | 137 | 33.8 | 268 | 66.2 |  | 1263 | 785 | 62.2 | 478 | 37.8 |  |  |
| **Below median** | | | | | | | | | | | | | | | | | | | |
| **Total** | 11450 | 8998 | 78.6 | 2452 | 21.4 | - | 14680 | 8882 | 60.5 | 5789 | 39.5 | - | 30893 | 25141 | 81.4 | 5752 | 18.6 |  |  |
| **TTS** |  |  |  |  |  |  |  |  |  |  |  |  |  |  |  |  |  |  |  |
| ≤30 days | 9000 | 7080 | 79.1 | 1882 | 20.9 | <.001 | 11618 | 7339 | 63.2 | 4279 | 36.8 | <.001 | 27678 | 22578 | 81.6 | 5100 | 18.4 | 0.03 |  |
| >30 days | 2450 | 1840 | 76.7 | 570 | 23.3 |  | 3062 | 1542 | 50.4 | 1519 | 49.6 |  | 3215 | 2563 | 79.7 | 652 | 20.3 |  |  |
| **Above median** | | | | | | | | | | | | | | | | | | | |
| **Total** | 10645 | 8657 | 79.4 | 2188 | 20.6 | - | 14528 | 9178 | 63.2 | 5350 | 36.8 | - | 27878 | 22806 | 81.8 | 5072 | 18.2 |  |  |
| **TTS** |  |  |  |  |  |  |  |  |  |  |  |  |  |  |  |  |  |  |  |
| ≤30 days | 9061 | 7241 | 79.9 | 1820 | 20.1 | <.001 | 13160 | 8410 | 63.9 | 4750 | 36.1 | <.001 | 25933 | 21298 | 82.1 | 4635 | 17.9 | <.001 |  |
| >30 days | 1584 | 1216 | 76.8 | 368 | 23.2 |  | 1368 | 768 | 56.1 | 600 | 43.9 |  | 1945 | 1508 | 77.5 | 437 | 22.5 |  |  |
| p<0.001^***^, p<0.01^**^, p<0.05^*^  Abbreviation: TTS: Time to surgery; SD: Standard Deviation | | | | | | | | | | | | | | | | | | | |
|  | | | | | | | | | | | | | | | | | | | |
