# Supplementary material for: The delayed cancer treatment and economic inequality in Korea: results of common cancers by the time-to-surgery
Source: Epidemiol Health. 2025 Sep 27;47:e2025056. doi: 10.4178/epih.e2025056 (PMC12869139; doi:10.4178/epih.e2025056)
Supplement: Supplementary Material 10. — Characteristics of the study population based on income level, LOS, and Medical costs [file epih-47-e2025056-Supplementary-10.docx]

| **Supplementary Material 10. Characteristics of the study population based on income level, LOS, and Medical costs** | | | | | | | | | | | | | |
| --- | --- | --- | --- | --- | --- | --- | --- | --- | --- | --- | --- | --- | --- |
| **Variable** | **LOS (Unit: days)** | | | | | | **Medical costs (Unit: 1,000,000￦)** | | | | | | |
|  | **Lung cancer** | | **Liver cancer** | | **Colorectal cancer** | | **Lung cancer** | | **Liver cancer** | | **Colorectal cancer** | | |
|  | **Mean (±SD)** | **p-value** | **Mean (±SD)** | **p-value** | **Mean (±SD)** | **p-value** | **Mean (±SD)** | **p-value** | **Mean (±SD)** | **p-value** | **Mean (±SD)** | **p-value** |  |
| **Medical aid** | | | | | | | | | | | | | |
| **Total** | 151.2 (266.5) |  | 195.1 (287.3) |  | 204.2 (370.6) |  | 44.4 (51.6) |  | 58.7 (56.9) |  | 42.4 (49.9) |  |  |
| **TTS** |  |  |  |  |  |  |  |  |  |  |  |  |  |
| ≤30 days | 140.6 (210.4) | <.001 | 185.7 (270.6) | <.001 | 195.7 (363.4) | <.001 | 34.1 (48.2) | <.001 | 51.5 (60.4) | <.001 | 34.3 (46.2) | <.001 |  |
| >30 days | 153.9 (215.3) |  | 215.2 (312.4) |  | 225.3 (396.4) |  | 63.2 (60.5) |  | 66.2 (73.2) |  | 53.7 (52.8) |  |  |
| **Below median** | | | | | | | | | | | | | |
| **Total** | 97.5 (193.9) |  | 141.2 (231.8) |  | 112.2 (226.9) |  | 42.7 (52.7) |  | 64.5 (80.6) |  | 40.3 (50.9) |  |  |
| **TTS** |  |  |  |  |  |  |  |  |  |  |  |  |  |
| ≤30 days | 90.3 (185.4) | <.001 | 135.2 (224.1) | <.001 | 108.6 (225.5) | <.001 | 39.5 (47.3) | <.001 | 61.7 (75.9) | <.001 | 38.5 (45.9) | <.001 |  |
| >30 days | 115.6 (215.9) |  | 164.6 (253.3) |  | 128.3 (241.5) |  | 45.8 (56.2) |  | 72.7 (85.5) |  | 42.1 (60.4) |  |  |
| **Above median** | | | | | | | | | | | | | |
| **Total** | 82.1 (174.1) |  | 123.1 (195.6) |  | 101.5 (224.4) |  | 38.5 (49.4) |  | 62.6 (74.3) |  | 36.2 (47.5) |  |  |
| **TTS** |  |  |  |  |  |  |  |  |  |  |  |  |  |
| ≤30 days | 78.8 (170.6) | <.001 | 118.2 (190.0) | <.001 | 100.1 (217.0) | <.001 | 36.2 (43.8) | <.001 | 54.5 (63.7) | <.001 | 35.6 (44.3) | <.001 |  |
| >30 days | 103.7 (202.2) |  | 138.7 (222.8) |  | 123.7 (230.5) |  | 49.6 (58.1) |  | 65.6 (82.3) |  | 49.2 (58.1) |  |  |
| p<0.001^***^ , p<0.01^**^, p<0.05^*^ Abbreviation: LOS: Length of Stay; TTS: Time to surgery; SD: Standard Deviation | | | | | | | | | | | | | |
